# Lactate dehydrogenase is not a useful marker for relapse in patients on surveillance for stage I germ cell tumours

**DOI:** 10.1038/sj.bjc.6603087

**Published:** 2006-04-11

**Authors:** C Ackers, G J S Rustin

**Affiliations:** 1 Mount Vernon Hospital, Rickmansworth Road, Northwood, Middlesex HA6 2RN, UK

**Keywords:** LDH, relapse, germ cell tumours, surveillance

## Abstract

As part of surveillance protocols for stage I germ cell tumours, many centres routinely measure human chorionic gonadotrophin (HCG), alpha feto-protein (AFP) as well as lactate dehydrogenase (LDH). In conjunction with regular imaging and clinical examination, does routine measurement of LDH add anything to our relapse/pick up rate? Records of 494 patients at Mount Vernon Hospital who relapsed on surveillance between 1985 and 2005 were examined. Of the 494 patients who relapsed, 125 had raised LDH at the time of relapse. 112 of these had a concurrent rise in either AFP, HCG or both, 11 had their disease detected on CT before the rise in LDH, one had a clinically palpable para-aortic mass and the final patient complained of back pain and his retroperitoneal disease was thus discovered on imaging. Routine measurement of LDH in patients on surveillance for stage I germ cell tumours does not add to the early detection of relapse.

A high proportion of patients with stage I testicular germ cell tumours are managed by close surveillance post-orchidectomy (Read *et al*, 1992). The surveillance protocol consists of regular physical examinations, chest X-rays, CT scans and measurement of tumour markers. All protocols include measurement of human chorionic gonadotrophin (HCG) and alpha feto-protein (AFP). Although lactate dehydrogenase (LDH) is elevated in more than 50% of patients with germ cell tumours and is an important prognostic indicator (Group IGCU, 1997), it is not clear whether it is valuable in surveillance. The majority of patients in our surveillance programme at Mount Vernon Hospital have had regular LDH measurements and we have therefore performed a retrospective analysis to determine whether regular measurements of LDH lead to earlier diagnosis of relapse than that discovered by the other components of the surveillance protocol.

## MATERIALS AND METHODS

The notes, blood results and imaging of 494 patients with stage I germ cell tumours who relapsed while on surveillance at Mount Vernon Hospital between 1985 and 2005 were reviewed.

The method(s) used to identify relapse was identified to ascertain how often a rise in LDH helped to detect relapse.

## RESULTS

The majority of patients had nonseminomatous germ cell tumours (399 patients (81%)) and the remainder were seminomas (95 patients (19%)). A total of 125 had a raised LDH at the time of relapse, but 112 had a concurrent rise in either AFP, HCG or both. Of the other 13, 11 were seminomas and disease recurrence had already been detected on routine surveillance CT scans before the rise in LDH. Of the remaining two patients, disease relapse was identified clinically alongside the rise in LDH. One patient had a palpable para-aortic mass, the other complained of back pain and para-aortic disease was subsequently identified on imaging. There were no cases in which a rise in LDH alone was the only marker of disease relapse.

During follow-up, a further 26 patients had a benign cause for a rise in their LDH. Eighteen were owing to intercurrent infections and the LDH normalised spontaneously. One had a familial, persistently raised LDH. The final seven had an acute rise in LDH owing to heavy alcohol intake just before their blood test. Repeat samples within 1 week were entirely normal.

## DISCUSSION

The level of serum LDH has independent prognostic significance in patients with advanced germ cell tumours, reflected within the IGCCCG grouping. Increases in the serum concentration are a reflection of tumour burden, growth rate and cellular proliferation.

Lactate dehydrogenase is comprised of multiple isoenzymes, but in practice, the combined isoenzyme value is used for clinical decision making (Von Eyben, 2001). Increased serum LDH concentrations are observed in approximately 60% of NSGCTs with advanced disease and in 80% of patients with advanced seminoma.

In a paper published in Cancer (Lippert and Javadpour, 1981), LDH in 80 patients with testicular germ cell tumours was reviewed. Serum LDH was found to be elevated more frequently with increasing tumour bulk (78% of patients with stage III disease and only 26% of stage II patients). It may therefore be that with vigilant surveillance, relapses are detected at a very early stage and tumour bulk may be minimal. Hence a rise in LDH is less commonly seen.

## CONCLUSION

Routine measurement of LDH in patients on surveillance for stage I germ cell tumours does not add to the early detection of relapse.

Lactate dehydrogenase remains important in determining prognosis.
